# New approaches to the analysis of eye movement behaviour across expertise while viewing brain MRIs

**DOI:** 10.1186/s41235-018-0097-4

**Published:** 2018-04-25

**Authors:** Emily M. Crowe, Iain D. Gilchrist, Christopher Kent

**Affiliations:** School of Experimental Psychology, University of Bristol, 12a Priory Road, Bristol, BS8 1TU UK

**Keywords:** Brain tumour detection, Eye-tracking, ScanMatch, Expertise, Magnetic resonance imaging, Medical image perception

## Abstract

Brain tumour detection and diagnosis requires clinicians to inspect and analyse brain magnetic resonance images. Eye-tracking is commonly used to examine observers’ gaze behaviour during such medical image interpretation tasks, but analysis of eye movement sequences is limited. We therefore used ScanMatch, a novel technique that compares saccadic eye movement sequences, to examine the effect of expertise and diagnosis on the similarity of scanning patterns. Diagnostic accuracy was also recorded. Thirty-five participants were classified as Novices, Medics and Experts based on their level of expertise. Participants completed two brain tumour detection tasks. The first was a whole-brain task, which consisted of 60 consecutively presented slices from one patient; the second was an independent-slice detection task, which consisted of 32 independent slices from five different patients. Experts displayed the highest accuracy and sensitivity followed by Medics and then Novices in the independent-slice task. Experts showed the highest level of scanning pattern similarity, with medics engaging in the least similar scanning patterns, for both the whole-brain and independent-slice task. In the independent-slice task, scanning patterns were the least similar for false negatives across all expertise levels and most similar for experts when they responded correctly. These results demonstrate the value of using ScanMatch in the medical image perception literature. Future research adopting this tool could, for example, identify cases that yield low scanning similarity and so provide insight into why diagnostic errors occur and ultimately help in training radiologists.

## Significance

According to the American Brain Tumor Association (2017), nearly 80,000 cases of primary brain tumour are expected to be diagnosed in 2017. Clearly, the successful detection of brain tumours is essential for diagnosis, patient monitoring, treatment planning and patient prognosis. Current best practice requires clinicians to inspect and analyse MRIs. Eye-tracking has commonly been used to examine the gaze behaviour of observers in this task, but limited research has examined the sequence of eye movements observers engage in when searching for abnormalities. We used a novel technique, ScanMatch, to compare saccadic eye movement sequences in a brain tumour detection task. This method utilises both temporal and spatial components of eye movement sequences and therefore enables a more detailed investigation into the search behaviour of observers. This research demonstrates the effective application of ScanMatch to the medical image perception literature thus offering a new approach to the analysis of eye movement behaviour.

## Background

Medical imaging is a crucial tool when making diagnostic and treatment decisions. Clinicians inspect an image to first detect and then interpret any abnormalities in the context of a given medical problem. Approximately 5 billion diagnostic examinations are performed worldwide each year (Ciarrapico et al., 2017), with radiologic image perception and interpretation occurring at a rate of more than one per second in the United States (Beam, Krupinski, Kundel, Sickles, & Wagner, 2006). Despite advances in computer-aided detection (CAD), final medical decision-making resides with clinicians and so is constrained by their perceptual and cognitive abilities. A large body of eye-tracking research has been conducted to better understand how clinicians engage in these interrelated processes and so provide insight into the relationship between visual search and diagnostic decision-making (Reingold & Sheridan, 2011).

Novice-expert studies, which examine the effect of expertise on gaze behaviour, have revealed that experts have faster overall search times to detect and confirm the presence of an abnormality (Krupinski, 1996; Krupinski et al., 2006). Experts fixate on lesions, or other regions of interest, faster and for longer than Novices (Kundel, Nodine, Conant, & Weinstein, 2007; Nodine, Kundel, Lauver, & Toto, 1996) and, in clear images, spend more time fixating regions that are most likely to contain abnormalities (Kundel, 1974). Nodine et al. (1996) suggested that Novices engage in less efficient searches as indexed by their greater coverage of the medical image (Krupinski, 1996; Manning, Ethell, Donovan, & Crawford, 2006; Nodine et al., 1996). Such research demonstrates that gaze behaviour changes as a function of expertise and hints at the possibility that we may be able to identify what characterises expertise and how knowledge of this can be used to improve training practices, and efficiency, for future clinicians.

Clinicians make diagnostic mistakes, with estimates suggesting approximately 30% false-negative and false-positive rates in radiology (Krupinski, 2010). The *prevalence effect* reveals that observers often miss rare targets, which are not often encountered in daily medical screening, compared to more frequently encountered targets (Evans, Tambouret, Evered, Wilbur, & Wolfe, 2011; Wolfe et al., 2007). Berbaum et al. (2001) revealed that radiologists are susceptible to satisfaction of search, whereby detection of a first abnormality detracts for the detection of subsequent abnormalities. Drew, Võ, and Wolfe (2013) demonstrated inattentional blindness in radiologists who failed to report seeing a gorilla within a lung-nodule detection task. Eye-tracking has therefore been used to investigate why and where errors occur. Kundel, Nodine, and Carmody (1978) developed a nodule detection model based on the assumption that prolonged dwell times indicate intensive processing of visual data to enable classification of false-negative responses to pulmonary nodules into different types of error. Scanning errors reflect a failure to fixate the lesion areas and recognition errors occur when the lesion area has been fixated but an observer does not detect the lesion. Decision-making errors are those where the interpretation of a lesion is incorrect. Out of 20 false-negative diagnoses performed by four radiologists, 30%, 25% and 45% were classified as scanning, recognition and decision-making errors, respectively (Kundel et al., 1978). Several researchers have reported longer fixations for false negatives (Kundel & Nodine, 2004; Nodine, Mello-Thoms, Kundel, & Weinsten, 2002), indicative of prolonged visual attention. Krupinski (2005) examined the effect of lesion subtlety on gaze behaviour and found that when subtler lesions were detected, dwell time was longer than for both the more obvious lesions and false negatives. Taken together these findings demonstrate a relationship between gaze behaviour and diagnostic accuracy, with certain behaviours characterising certain responses.

Scan paths which can capture both temporal and spatial components of an individual’s search have also been investigated in the medical image perception literature. Kundel and Nodine (2004) revealed that quantitative parameters derived from scan paths can be used to separate mammographers and trainee mammographers. Gandomkar, Tay, Brennan, and Mello-Thoms (2017) developed a model with 86.3% and 85.2% sensitivity and specificity, respectively, that distinguished expert and less experienced radiologists based on the spatial dynamics of their eye movements. Such research indicates that gaze behaviours characterise expertise in medical image interpretation tasks. Litchfield, Ball, Donovan, Manning, and Crawford (2008, 2010) revealed that viewing another person’s eye movements on a lung nodule detection task can improve performance while Sridharan, Bailey, McNamara, and Grimm (2012) reported higher sensitivity and specificity when Novices used a subtle gaze direction (SGD) technique that actively guides Novices along the scan path of an expert. Taken together, these studies indicate that visual guidance aids medical image interpretation.

Research also suggests that scanning patterns are related to diagnostic accuracy. Davies et al. (2016) examined how practitioners perceived electrocardiograms (ECGs) and determined whether visual behaviour can indicate differences in interpretation accuracy. Their results demonstrated a difference in the gaze behaviour between correct and incorrect interpretations of various heart-related measurements (e.g. identifying hyperkalaemia, torsades de pointes and atrial flutters) and so highlighted this as a factor in interpretation accuracy. Voisin, Pinto, Morin-Ducote, Hudson, and Tourassi (2013) used machine learning to successfully predict radiologists’ errors during the diagnosis of mammographic lesions by merging their gaze behaviour and textural characteristics of the image. Tourassi, Mazurowski, Harrawood, and Krupinski (2010) examined the potential of a context-sensitive computer-assisted detection (CADe) system that is guided by the user’s focus of attention. The context-sensitive mode of the system, which analysed radiologists scanning patterns and diagnostic decisions while inspecting 20 mammograms, reduced radiologists’ perceptual and cognitive errors in the diagnostic interpretation of screening mammograms more effectively than the conventional CADe system. Taken together, the eye-tracking literature indicates that scanning patterns are related to diagnostic accuracy.

However, only a limited amount of research has investigated similarities between the scan paths of participants viewing medical images. Wooding, Roberts, and Phillips-Hughes (1999) reported that trainee radiologists (16.5 months experience) showed the least within-group consistency compared to laymen (0 months experience), novices (2.3 months experience) and radiologists (90 months experience). Trainee radiologists also showed the least amount of similarity to radiologists compared to all other comparison groups. The authors suggested that trainees go through a developmental phase characterised by idiosyncratic patterns of attention allocation and eye movements. Leong, Nicolaou, Emery, Darzi, and Yang (2007) examined whether experience improves the consistency of visual search behaviour in fracture identification in plain radiographs. Using Kullback-Leibler divergence and Gaussian mixture model fitting, these authors reported that experts exhibited higher consistency in their search patterns.

The present study will extend the limited literature examining the similarity of scan paths. More specifically, we use ScanMatch (Cristino, Mathôt, Theeuwes, & Gilchrist, 2010), a well-established approach to quantifying the similarity between scanning patterns of individuals. At its core, ScanMatch is based on the Needleman-Wunsch algorithm, used commonly for comparing DNA sequences. We chose this method because it accounts for the temporal, spatial and sequential components of fixations and so overcomes limitations of existing string edit methods (Cristino et al., 2010). Moreover, the substitution matrix allows researchers to encode information about the relationship between specific regions of interest and so can account for semantic information as well (Cristino et al., 2010). Madsen, Larson, Loschky, and Rebello (2012) used ScanMatch to examine differences in the eye movements of individuals answering physics questions to examine if there was a difference for correct versus incorrect answers. This method has also been used to examine differences in scanning patterns between Novices and Experts evaluating paintings (Pihko et al., 2011) and while viewing surgical procedures (Kübler, Eivazi, & Kasneci, 2015), problem-solving (Nyamsuren & Taatgen, 2013), face-processing (Chaby, Hupont, Avril, Luherne-du Boullay, & Chetouani, 2017), and decision-making (Zhou et al., 2016). Anderson, Anderson, Kingstone, and Bischof (2015) compared the ability of several scan path comparison methods to reveal similarities both within and between individuals looking at natural scenes and concluded that ScanMatch is a remarkable improvement on more simple method string-edit and linear distance methods. Using this tool, we will investigate the effect of expertise and diagnosis on the similarity of scanning patterns in a brain tumour detection task using MRIs.

Medical image perception tasks include both static two-dimensional (2D) image viewing and dynamic stack viewing. Stack viewing involves a clinician quickly scrolling through a stack of 2D images to get a three-dimensional (3D) impression of the anatomical structure of an organ (Nakashima, Komori, Maeda, Yoshikawa, & Yokosawa, 2016). The shift from static to dynamic viewing has changed the task of medical image interpretation with a tiled set of 2D images containing less information than a volumetric image (Krupinski et al., 2012). Medical students tend to perform worse on volumetric images than on 2D images (Ravesloot, van der Gijp et al., 2015; Ravesloot, Van Der Schaaf et al., 2015). van der Gijp et al. (2015) showed that radiology clerks take more time, and engage in more and different cognitive processes, when interpreting volumetric images than 2D images with Stuijfzand et al. (2016) reporting an effect of image information (i.e. 2D or 3D) on self-reported mental effort used to index cognitive load. 3D volumetric image interpretation better reflects the clinical setting for inspecting brain MRI images in which the brain is separated into cross-sections or ‘slices’. Therefore, in this experiment, it is important to investigate eye-gaze behaviour in dynamic viewing and the visual search of clinicians viewing sequentially presented, *dependent*, medical images (Drew, Võ, Olwal et al., 2013; Nakashima et al., 2016). The dependence between sequential images from the same brain is important as the clinician may use information from previous slices to direct their attention on the current slice.

Despite the wealth of research into medical image interpretation, most studies have used either the chest or breast as stimuli (see Reingold & Sheridan, 2011, for a review). Ostrom et al. (2015) predicted that in 2016 approximately 77,670 primary brain and central nervous system tumours were expected to be diagnosed in the United States. Although eye-tracking-based research has assessed clinician’s inspection of brain MRI images for glioma diagnosis (Cavaro-Ménard, Tanguy, & Le Callet, 2010) and the eye-gaze distribution of neurologists when viewing CT images of stroke patients (Matsumoto et al., 2011), there is significantly less work examining how clinicians and novices view MRI images of the brain. To start to address this gap, the current study uses MRI brain images as stimuli.

Here, we use a novice-expert design to examine how eye-gaze parameters change across three expertise levels (i.e. undergraduate students: *Novices*; third and fourth year undergraduate medical students: *Medics*; and medical professionals: *Experts*) in a brain tumour detection task using MRIs. This study is the first to apply ScanMatch to the medical image perception literature to better understand the temporal dynamics of image interpretation. Thirty-five participants completed both a whole-brain (Experiment 1a) and independent-slice (Experiment 1b) brain tumour detection task to further investigate a prevalent issue in the literature, namely the effect of viewing modality on visual search and performance. In the whole-brain task, eye-gaze data was recorded while participants sequentially viewed 60 slices of a patient’s brain MRI and in the independent-slice task, both eye-gaze data and performance measures were recorded when participants inspected 32 brain MRIs (16 tumorous; 16 healthy). This exploratory work examines the efficacy of a novel technique, ScanMatch, within the medical image perception literature. The application this technique could have practical implications including the development of medical training and monitoring of students’ acquisition of expertise.

## Experiment 1a: whole-brain

### Method

#### Participants

Thirty-five participants were recruited into three groups based on their level of expertise in brain MRI interpretation. The *Novice group* consisted of 18 undergraduates at the University of Bristol studying any subject apart from medicine, dentistry or veterinary sciences. The *Medic* group were ten medical students from the University of Bristol in either their third (*n* = 8) or fourth (*n* = 2) year of study. Seven *Experts* were recruited from a National Health Service (NHS) hospital and consisted of trainee neuroradiologists (*n* = 3; mean experience 2.5 years), consultant neuroradiologists (n = 2; mean experience 8 years) and consultant neurologists (n = 2; mean experience 12 years). All participants had normal or corrected-to-normal vision and gave written informed consent in accordance with the Declaration of Helsinki (2008). Ethical approval was obtained from the Faculty of Science Human Research Ethics Committee at the University of Bristol.

#### Stimuli and apparatus

**Fig. 1:**
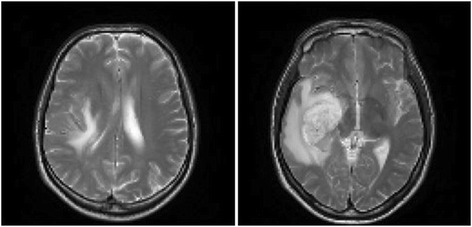
Example slices from the entire brain set used in the stack viewing task, Experiment 1a

All brain images and diagnoses were obtained from a UK NHS hospital. Stimuli for the whole-brain task consisted of 60 T2 brain MRI images from one patient with a right medial temporal lobe intrinsic tumour (see Fig. 1 for example slices). T1 and T2 images are commonly acquired medical images used in clinical settings for inspecting brains. We used T2 images for the whole-brain task because this is most heavily relied upon for brain tumour detection. All stimuli were registered using SPM5 (Penny et al., 2001). Stimuli were presented using a custom-made programme written using MATLAB (The MathWorks, Inc., 2013) and the Psychophysics Toolbox (Psychtoolbox-3; Brainard, 1997; Kleiner, Brainard, & Pelli, 2007; Pelli, 1997). Eye-gaze data were recorded from the participant’s dominant eye using the Eye-Link 1000 (SR Research, Mississauga, ON, Canada), an infrared tracking system that uses the pupil centre in conjunction with corneal to sample eye position at 1000 Hz. For each data sample, a dedicated parser algorithm (SR Research, Mississauga, ON, Canada) computes the instantaneous velocity and acceleration of the eye. These are then compared to threshold criteria for velocity (30°/s) and acceleration (8000°/s2). If either is above threshold, the eye movement is classified as a saccade. A MATLAB script (The MathWorks, Inc., 2013) was then used to extract all the saccades from the Eyelink Data File. Using a chin rest, participants viewed stimuli on a colour laptop monitor (1280 × 800 pixel resolution) from a distance of 50 cm in a darkened room.

#### Design

This was a mixed design with Expertise Level (Novice, Medic, Expert) as the between-subject factor and Run Through (Run 1, Run 2) as the within-subjects factor. In order to assess intra-group similarity in scanning patterns, within-group comparison was included as a between-subject factor (i.e. Novice-Novice, Medic-Medic, Expert-Expert). To investigate between-group similarity, between-group comparison was included as a within-subject factor (e.g. Novice-Novice, Novice-Medic, Novice-Expert).

#### Procedure

A standard nine-point calibration and validation procedure was performed in which observers were asked to fixate on a black cross that appeared randomly on a 3 × 3 grid. Following this, participants were instructed to search the stack of brain MRI images for any tumorous tissue. Each slice was presented for 1500 ms and was immediately followed by the subsequent slice. This process was completed twice with a 2000-ms break between runs. Participants were told to freely inspect the brain during both runs. The stimuli were selected so that the tumour was very clear to all observers and therefore participants were not required to provide a diagnosis (see Fig. 1). The task lasted approximately 4 min.

#### Analysis

**Fig. 2:**
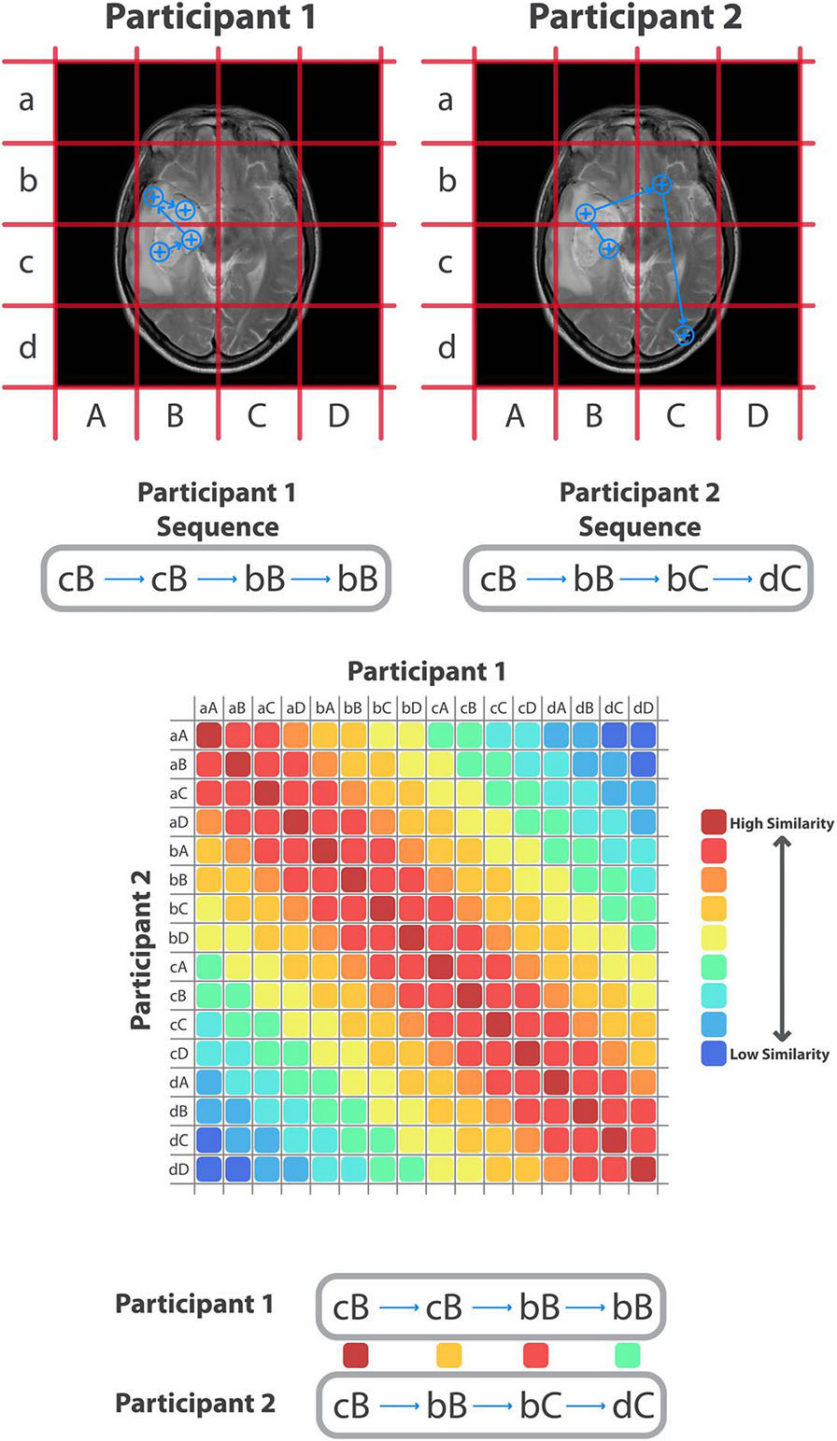
Simplified details of the ScanMatch method used to calculate similarity in scanning patterns. The top panel shows the fixations of two participants overlaid on an example stimulus. A grid-based region-of-interest (ROI) mask is also overlaid with a combination of lower- and upper-case letters used to define each ROI. The middle panel demonstrates the generation of letter sequences used to describe each participants’ search behaviour and the substitution matrix that provides information on the relationship between two ROIs. The bottom panel shows how two participants scanning behaviour are compared. The colour-coded substitution matrix is used to calculate an alignment score which indexes how similar two participants’ scanning behaviour is. Red squares indicate a high score because the two participants fixated in the same ROI whereas blue squares indicate a low score because the two participants fixated in different ROIs. Alignment scores are then normalised to generate a score between 0 (low similarity) to 1 (high similarity). See Cristino et al. (2010) for a full description of the ScanMatch method

Our analysis focused on the fixation patterns. We measured the similarity of the sequences of fixations using ScanMatch (Cristino et al., 2010). A letter-based string sequence was generated for each participant that described their fixations. The sequences of different participants are then compared using ScanMatch. An alignment score is generated and then normalised to provide a ScanMatch similarity score, namely an index of scanning similarity. A similarity score of 1 indicates that the sequences are identical while a score of 0 indicates that there is no similarity. Figure 2 shows details of the ScanMatch method. More specifically, within-group comparisons and between-group comparisons were examined for Run1 and Run 2. The sequence of fixations for Run 1 and Run 2 were those that were recorded during the first and second presentation of the 60 slices which constituted an entire brain, respectively. For each run, the sequence was presented across all slices, because the first fixation location on a given slice would have depended on the final fixation location of the previous slice. The sequence and duration of fixations were used to generate a letter-string sequence that corresponded to spatial locations. We then compared this sequence with other participants’ sequences.

**Fig. 3:**
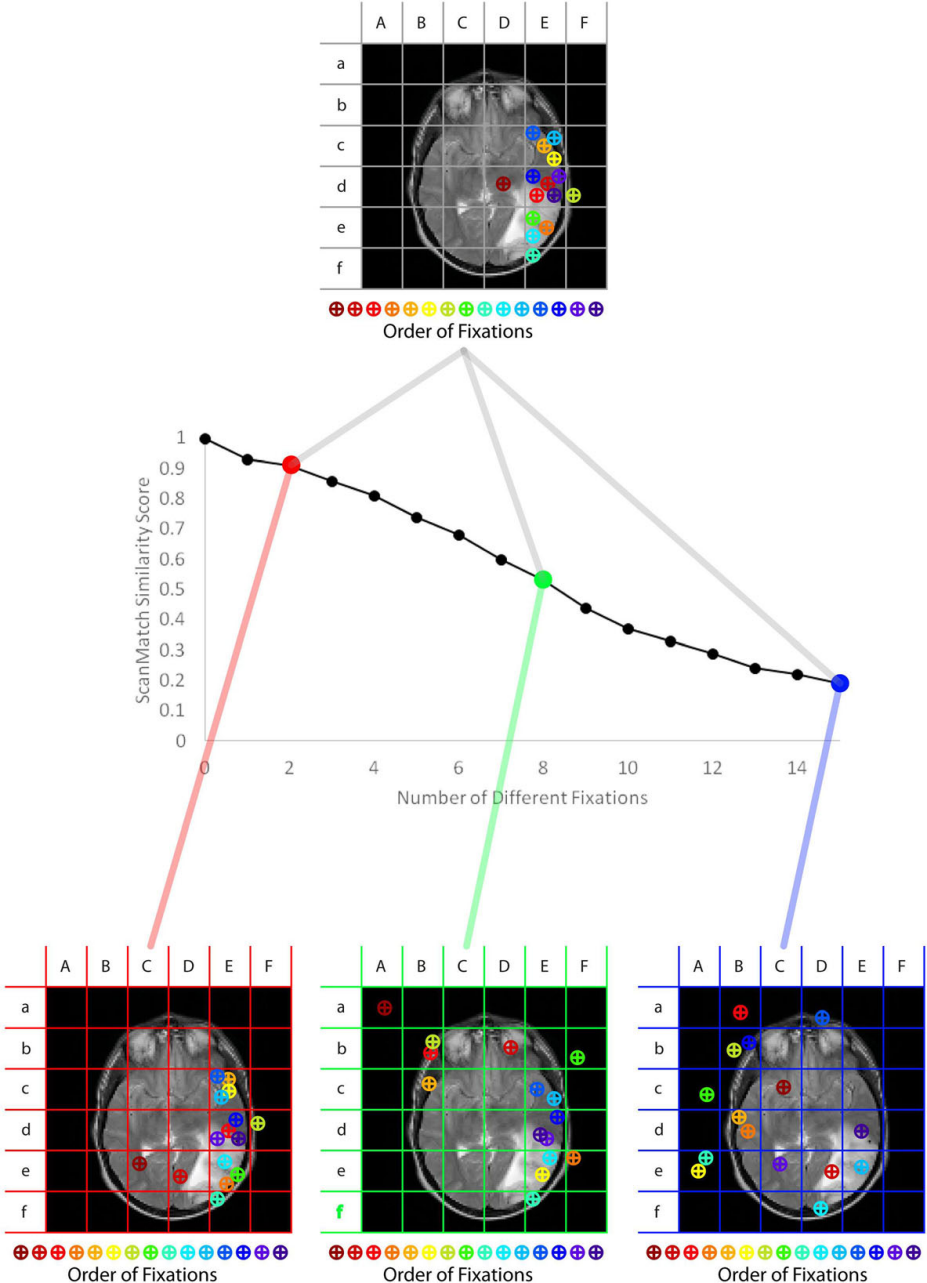
An illustration of the relationship between scanning sequences and ScanMatch similarity scores. The top panels show the scanning sequence of a Participant N (note fixations are plotted according to the correct ROIs not the specific fixation location for presentation purposes). The middle panel shows the effect number of different fixations between two sequences on ScanMatch similarity score. The bottom panel shows scanning sequences that have 2 (red), 8 (green) and 15 (blue) random fixations inserted into the scanning sequence of Participant N to show the effect of this on ScanMatch similarity score

Figure 3 explains how to interpret different ScanMatch similarity scores in the context of this experiment. In order to gain a clearer insight into this, we used the experimental data as the reference sequence and compared this with a test sequence. The first test sequence had one fixation replaced with a randomly selected alternative fixation. After each comparison, another random replacement was made in the same manner. This analysis demonstrates effect of the number of different fixations between two scanning sequences on ScanMatch similarity score (see Fig. 3). Of course, the actual pattern is more complicated than this as ScanMatch takes into account temporal order, so it does not simply reflect the number of fixation differences (this is a proxy in order to illustrate what a difference may mean in terms of fixation differences).

### Results

The results reported below lie in the middle range of ScanMatch similarity scores (see Fig. 3). This indicates that scanning patterns are not in high agreement nor entirely random. Moreover, while many of the reported differences between conditions are small, they are statistically reliable. A 3 × 2 mixed ANOVA revealed that scanning patterns were more similar for Run 1 (*M* = 0.56, 95% confidence interval [CI] = 0.55–0.57) than Run 2 (*M* = 0.52, 95% CI = 0.50–0.54), *F*(1,32) = 27.98, *p* < 0.001, *η*_*p*_^*2*^ = 0.466. An effect of within-group comparison on ScanMatch similarity scores was also found, *F*(1,32) = 6.86, *p* = 0.003, *η*_*p*_^*2*^ = 0.300. Experts displayed the most similar scanning patterns (*M* = 0.58, 95% CI = 0.55–0.61), followed by Novices (*M* = 0.54, 95% CI = 0.52–0.56) and then Medics (*M* = 0.50, 95% CI = 0.47–0.53). There was no interaction, *F*(2,32) = 1.72, *p* = 0.196, *η*_*p*_^*2*^ = 0.097.

A similar qualitative pattern of results was observed for between-group comparisons (see Table 1). Across all between-group comparisons, scanning similarity was higher for Run 1 than Run 2 (all *F* > 6.34, *p* < 0.014). There was an effect of between-group comparison when comparing novices against all other expertise levels *F*(2,34) = 13.10, *p* < 0.001, *η*_*p*_^*2*^ = 0.435. Novice-Novice and Novice-Expert comparisons were more similar than Novice-Medic comparisons. There was also an interaction, *F*(2,34) = 8.56, *p* = 0.001, *η*_*p*_^*2*^ = 0.335, with all between-group comparisons revealing higher scanning similarity for Run 1 than Run 2.

**Table 1:** Means (95% CI) for each between-group comparison and run through for ScanMatch similarity score

		Novice	Medic	Expert
Novice	Run 1	0.55 (0.53–0.58)	0.54 (0.52–0.57)	0.57 (0.54–0.60)
	Run 2	0.53 (0.50–0.56)	0.50 (0.47–0.52)	0.54 (0.50–0.57)
Medic	Run 1	0.54 (0.51–0.58)	0.53 (0.50–0.56)	0.54 (0.51–0.58)
	Run 2	0.51 (0.47–0.55)	0.47 (0.44–0.50)	0.50 (0.43–0.56)
Expert	Run 1	0.57 (0.54–0.60)	0.56 (0.53–0.59)	0.61 (0.60–0.62)
	Run 2	0.54 (0.49–0.58)	0.51 (0.48–0.53)	0.55 (0.52–0.58)

Medic-Medic comparisons were less similar than Medic-Novice comparison and Medic-Expert comparison, *F*(2,18) = 7.34, *p* = 0.008, *η*_*p*_^*2*^ = 0.413. Expert-Expert and Expert-Novice comparisons were more similar than Expert-Medic comparisons, *F*(2,12) = 15.83, *p* < 0.001, *η*_*p*_^*2*^ = 0.725. Study participants did not consent to data sharing so supporting data cannot be made available.

## Experiment 1b: independent-slices

Since most existing research uses 2D viewing modalities, we also examined the effect of expertise and diagnosis on eye-gaze behaviour and diagnostic accuracy in an independent-slice brain tumour detection task. This provides insight into the effect that viewing modality may have on gaze behaviour in such tasks.

### Method

#### Participants

All participants who completed Experiment 1a also participated in Experiment 1b.

#### Design

This was a mixed design with expertise level (Novice, Medic, Expert) as the between-subject factor and diagnosis (clear, tumorous) as the within-subject factor. For some analyses, we included stimulus-response classification (i.e. true positive, false negative, false positive, true negative) as the within-subject factor. In order to assess within-group similarity in scanning patterns, within-group comparison was included as a between-subject factor (i.e. Novice-Novice, Medic-Medic, Expert-Expert). To investigate between-group similarity, between-group comparison was included as a within-subject factor (i.e. each participant has a similarity score with all expertise groups).

#### Stimuli and apparatus

**Fig. 4:**
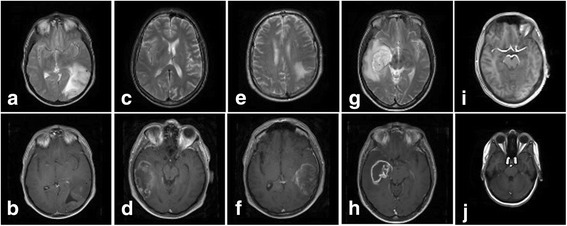
Example slices from the static viewing task. Images (**a**) (T2) and (**b**) (T1) are from patient 1, (**c**) (T2) and (**d**) (T1) are from patient 2, (**e**) (T2) and (**f**) (T1) are from patient 3, (**g**) (T2) and (**h**) (T1) are from patient 4 and (**i**) (T2) and (**j**) (T1) from are from patient 5

Figure 4 shows the stimuli for the static viewing task consisted of 32 images from five patients (four unhealthy; one healthy). Four slices (two T1; two T2) were used as stimuli from each unhealthy patients’ scan and so the conspicuity varied (by-item accuracy: *M* = 79%, *95% CI* 72–85%, with three cases at ceiling [i.e. 100%]) dependent upon the location within the scan from which the slice was taken. Sixteen slices (eight T1; eight T2) were used as stimuli from the healthy patients’ scan. Registration and presentation of stimuli was the same as reported for Experiment 1a and the same apparatus was used.

#### Procedure

Participants completed this task straight after the stack viewing task. Participants were instructed to inspect the images freely and decide whether they believed a tumour was present or absent. Each trial started with a fixation cross in the centre of the screen upon which participants had to fixate for the test stimuli to appear. Test stimuli were presented for 5 s after which participants had to provide a diagnosis (i.e. tumorous or clear) and confidence rating (in this case: Guess, Maybe, Probably, Definitely). All participants were instructed to freely inspect the images and then provide a diagnostic decision by moving a mouse over a centrally located array which had the response options within. Participants were not required to localise the tumour because we did not want the response procedure to interfere with scanning patterns. The testing session took approximately 40 min.

#### Analysis

Accuracy and confidence ratings were recorded and we calculated standard signal detection statistics *d’*, which indexes observer’s ability to distinguish between tumorous (target-present) and clear (target-absent) images, and *C*, which gives a measure of an observer’s bias to respond positively or negatively to an image. As with Experiment 1a, ScanMatch similarity scores were calculated. We calculated scores using both the actual diagnosis (i.e. tumour-present or absent) and the four categories of the stimulus-response matrix (true positive, false negative, false positive, true negative) as independent variables to fully reflect the diagnostic decision of each participant.

### Results

Table 2 shows the mean and standard deviation for the performance measures. There was an effect of expertise level on accuracy, *F*(1,32) = 15.42, *p* < 0.001, *η*_*p*_^*2*^ = 0.491. Experts were more accurate than Medics who were more accurate than Novices. Expertise level also affected confidence ratings, *F*(1,32) = 20.28, *p* < 0.001, *η*_*p*_^*2*^ = 0.559. Experts were more confident than Medics who were more confident than Novices. Participants reported higher confidence for tumorous compared to clear images, *F*(1,32) = 15.47, *p* < 0.001, *η*_*p*_^*2*^ = 0.326. There were no other effects on accuracy or confidence (all *F* < 1.51, *p* > 0.236). There was an effect of expertise level on discriminability, *F*(2, 34) = 19.34, *p* < 0.001, *η*_*p*_^*2*^ = 0.547. Medics were more sensitive than Novices and Experts were more sensitive than Medics. There was no effect of expertise level on bias, *F*(2, 34) = 1.78, *p* = 0.185, *η*_*p*_^*2*^ = 0.100.

**Table 2:** Mean and standard deviations (in parentheses) for performance measures in Experiment 1B

	Accuracy		Confidence		*d’*	*C*
	Tumour	Clear	Tumour	Clear		
Novice	0.68 (0.13)	0.79 (0.13)	1.47 (0.46)	1.29 (0.48)	1.43 (0.53)	0.20 (0.35)
Medic	0.81 (0.12)	0.84 (0.18)	2.07 (0.25)	1.88 (0.42)	2.09 (0.58)	0.08 (0.48)
Expert	0.95 (0.04)	0.90 (0.12)	2.61 (0.35)	2.15 (0.44)	2.88 (0.47)	0.13 (0.30)

We first analysed the results using a 3 (within-group comparison: Novice-Novice, Medic-Medic, Expert-Expert) × 2 (diagnosis: tumorous, clear) by within-group comparison and diagnosis (i.e. tumorous or clear). A main effect of within-group comparison, *F*(1,32) = 12.88, *p* < 0.001, *η*_*p*_^*2*^ = 0.446, was revealed. Medics (*M* = 0.51, 95% CI = 0.48–0.54) had less similar scanning patterns than both Novices (*M* = 0.55, 95% CI = 0.53–0.57) and Experts (*M* = 0.58, 95% CI = 0.57–0.59). There was also a main effect of diagnosis, *F*(1, 32) = 4.59, *p* = 0.040, *η*_*p*_^*2*^ = 0.125. Scanning patterns were more similar for clear (*M* = 0.55, 95% CI = 0.54–0.56) than tumorous images (*M* = 0.54, 95% CI 0.53–0.55). There was no interaction, *F*(2, 32) = 0.19, *p* = 0.829, *η*_*p*_^*2*^ = 0.012.

**Fig. 5:**
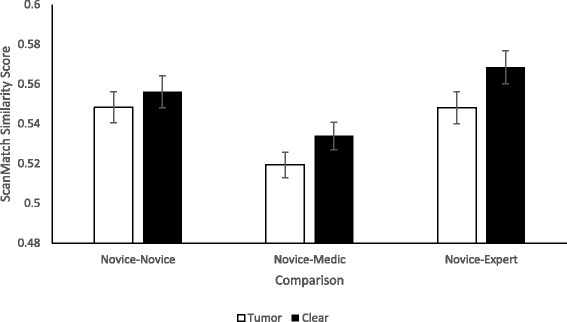
Interaction between between-group comparison and diagnosis on ScanMatch similarity score when Novices are the reference group. Error bars represent standard error

Table 3 shows the between-group comparison for each diagnosis. Three 3 × 2 repeated measures ANOVAs were conducted to examine between-group similarity in scanning patterns (e.g. Novice-Novice; Novice-Medic; Novice-Expert). When comparing Novices to all other expertise levels there was a main effect of between-group comparison, *F*(2,34) = 78.75, *p* < 0.001, *η*_*p*_^*2*^ = 0.822. Novices had less similar scanning patterns to Medics than to themselves and Experts. There was an effect of diagnosis, *F*(1,37) = 4.98, *p* = 0.039, *η*_*p*_^*2*^ = 0.227. Scanning patterns were more similar for clear compared with tumorous images. There was also an interaction, *F*(2,34) = 12.06, *p* < 0.001, *η*_*p*_^*2*^ = 0.541, with scanning patterns being most similar for clear images across all between-group comparisons (see Fig. 5).

**Table 3:** Means (95% CI) for each between-group comparison and actual diagnosis for ScanMatch similarity scores

		Novice	Medic	Expert
Novice	Tumorous	0.55 (0.53–0.57)	0.52 (0.51–0.53)	0.55 (0.53–0.57)
	Clear	0.56 (0.54–0.57)	0.53 (0.52–0.55)	0.57 (0.55–0.59)
Medic	Tumorous	0.53 (0.48–0.58)	0.50 (0.47–0.53)	0.52 (0.48–0.56)
	Clear	0.54 (0.49–0.58)	0.51 (0.48–0.54)	0.53 (0.49–0.58)
Expert	Tumorous	0.55 (0.54–0.56)	0.53 (0.52–0.54)	0.57 (0.56–0.59)
	Clear	0.57 (0.56–0.58)	0.54 (0.53–0.55)	0.59 (0.57–0.60)

We also examined differences in Medics scanning patterns compared to themselves, Novices and Experts. There was a main effect of between-group comparison, *F*(2,18) = 12.33, *p* < 0.001, *η*_*p*_^*2*^ = .578. Medics were less similar in their scanning patterns to themselves than to Novices and Experts. There was no effect of diagnosis, *F*(1,9) = 2.46, *p* = 0.151, *η*_*p*_^*2*^ = 0.215, and no interaction between diagnosis and between-group comparison, *F*(2, 18) = 2.75, *p* = 0.091, *η*_*p*_^*2*^ = 0.234.

**Fig. 6:**
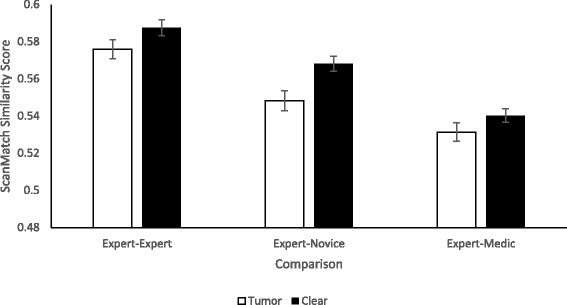
Interaction between between-group comparison and diagnosis on ScanMatch similarity score when Experts are the reference group. Error bars represent standard error

Using Experts as the reference, there was a main effect of between-group comparison, *F*(2,12) = 91.97, *p* < 0.001, *η*_*p*_^*2*^ = 0.939. Experts were more similar to themselves than Novices and Medics. There was a main effect of diagnosis, *F*(1,6) = 8.30, *p* = 0.028, *η*_*p*_^*2*^ = 0.580. Scanning patterns were more similar for clear compared to tumorous images. There was an interaction, *F*(2, 12) = 9.44, *p* = 0.003, *η*_*p*_^*2*^ = 0.611, with scanning patterns being most similar for clear images across all comparisons (see Fig. 6).

**Fig. 7:**
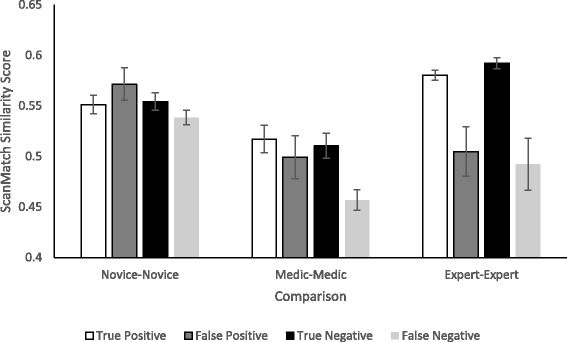
Interaction between within-group comparison and stimulus-response classification on ScanMatch similarity score. Error bars represent standard error

A 3 × 4 mixed ANOVA was used to examine differences in the scanning patterns within each expertise group and the effect of stimulus-response classification (i.e. true positive, false positive, true negative, false negative) on scanning pattern similarity. There was a main effect of within-group comparison, *F*(2,26) = 8.74, *p* = 0.001, *η*_*p*_^*2*^ = 0.402. Medics engaged in less similar scanning patterns with each other (*M* = 0.50, 95% CI = 0.47–0.53) than Novices (*M* = 0.55, 95% CI = 0.52–0.58) and Experts (*M* = 0.55, 95% CI = 0.50–0.60). There was also a main effect of stimulus-response category, *F*(3,78) = 7.28, *p* = 0.002, *η*_*p*_^*2*^ = 0.219. Scanning patterns were less similar for false negatives (*M* = 0.51, 95% CI = 0.49–0.53) than true positives (*M* = 0.54, 95% CI = 0.53–0.55), false positives (M = 0.54, 95% CI = 0.52–0.56) and true negatives (*M* = 0.55, 95% CI = 0.53–0.57). There was some evidence for an interaction, *F*(6,78) = 2.38, *p* = 0.063, *η*_*p*_^*2*^ = 0.155. Figure 7 shows that Experts were more similar in their scanning patterns for true positives (*M* = 0.58, 95% CI = 0.57–0.59) and true negatives (*M* = 0.59, 95% CI = 0.58–0.60), namely on correct trials, than false positives (*M* = 0.50, 95% CI = 0.45–0.55) and false negatives (*M* = 0.49, 95% CI = 0.44–0.54). Medics were less similar for false negatives (*M* = 0.46, 95% CI = 0.44–0.48) than true positives (*M* = 0.52, 95% CI = 0.51–0.53), false positives (*M* = 0.50, 95% CI = 0.46–0.54) and true negatives (*M* = 0.51, 95% = 0.49–0.53). It was not possible to examine between-group similarity in scanning patterns using stimulus-response classification because this requires two participants to provide the same response on a given stimuli which resulted in very small sample sizes.

Study participants did not consent to data sharing so supporting data cannot be made available.

### Discussion

This study examined the effect of expertise and diagnosis on performance and gaze behaviour in a brain tumour detection task using MRI scans. Novices, medical students and experts completed both a whole-brain tumour detection task, which better reflects the clinical setting, and an independent-slice brain tumour detection task commonly used in experimental research. In the independent task (Experiment 1b), diagnostic accuracy was recorded and revealed that Experts were the most accurate, confident and sensitive, followed by Medics and then Novices. This fits with the existing literature that diagnostic performance improves with expertise (Crowley, Naus, Stewart, & Friedman, 2003; Donovan & Litchfield, 2013) and confirms that participants were appropriately classified according to expertise level. A limitation of the task, however, was that participants were not required to localise the tumour and therefore could have been correct for the wrong reasons. Future research should collect localisation responses to provide more insight into the decision-making process. In addition, this result offers some insight into the development of expertise because three expertise levels were included. Medics, whose previous exposure to brain MRIs was to learn about brain structures and who had no formal training on the interpretation of brain MRIs, were more accurate and displayed higher sensitivity than Novices. We propose that their superior performance was driven by their knowledge of brain anatomy rather than perceptual skill. This fits with both Snowden, Davies, and Roling (2000) who reported that experience with a given stimulus enhances one’s sensitivity to critical features essential for detecting abnormalities and Nodine et al. (1999) who suggested that differences in resident performance resulted primarily from a lack of perceptual-learning experience. These findings indicate that perceptual skill is acquired with expertise but, since our Medics had little exposure to brain MRIs, it is unlikely that the perceptual component of medical image interpretation differentiated Novices’ and Medics’ performance on this task.

Since cross-sectional image interpretation now commonly uses volumetric images for diagnostic decision-making, we were interested in whether the same qualitative pattern of results was revealed when participants completed a whole-brain and independent-slice brain tumour detection task. This study was novel in examining eye-gaze behaviour in both viewing conditions which is a prevalent issue in the literature given the different demands of either tasks (Krupinski et al., 2012; Stuijfzand et al., 2016). A limitation of the whole-brain task was that each slice was presented for a fixed 1500-ms duration which does not reflect clinical practice in which clinicians can scroll freely through the image in any direction at their desired speed. Nevertheless, such dynamic stimulus presentations are still used within the literature (Nakashima et al., 2016). By controlling the presentation of stimuli, we were also able to directly compare Novices and Experts, which would not be possible if viewing times and styles varied greatly. The whole-brain task revealed that scanning patterns were more similar for Run 1 than Run 2. One interpretation is that scanning patterns in Run 1 were largely driven by saliency which supports models of visual saliency whereby fast and primitive bottom-up processes bias the observer towards the most salient stimuli in the early stages of exposure (Parkhurst, Law, & Niebur, 2002). In contrast, during Run 2, top-down processes such as prior experience (i.e. information from Run 1) and clinical knowledge guided visual attention in a goal-oriented manner that contributed to larger disparities in scanning patterns. We must acknowledge that the task given to participants, namely ‘*Search for a tumour*’ is much more well-defined than that experienced in a real clinical setting in which clinicians must search for various different diseases (e.g. haemorrhages) and therefore future research should try to replicate the clinical setting more closely.

The effect of expertise on scanning pattern similarity was consistent across both tasks. Experts engaged in the most similar inspection techniques, followed by novices and finally medical students (see Tables 1 and 3). This finding fits with both Leong et al. (2007) and Wooding et al. (1999) who reported that the most experienced observers showed greater consistency in their search patterns. Radiologists have undergone specific training and so acquired clinical knowledge that guides their search. More specifically, knowledge of the informative areas within an image and the probability distribution of areas likely to contain a tumour which increases the chances that they will fixate these regions in a similar order. We therefore propose that similar scanning patterns among experts are a consequence of specialised radiology training or expertise. Novices had no clinical knowledge of brain anatomy, so their searches were likely more driven by saliency cues such as asymmetry and contrast. This fits with research from standard visual search tasks in which visual features of an image, such as asymmetry, guide visual attention (Wolfe & Horowitz, 2004) and Matsumoto et al.’s (2011) finding that, when viewing brain computed tomography (CT) images, the areas novices frequently fixated often coincided with areas identified as outstanding in saliency maps. Medics had some clinical knowledge of the brain and so their search will have been driven by a combination of saliency cues and knowledge of brain anatomy. Several factors, such as age, nationality and sex, motivation and enthusiasm predict outcomes at medical school (Adam et al., 2015; Vaughan, Sanders, Crossley, O’neill, & Wass, 2015) and so it is likely that these medical students had different levels of clinical knowledge that contributed to more idiosyncratic scanning patterns. In line with Wooding et al. (1999), we propose that it is possible that the acquisition of expertise is not a smooth transition and that trainee radiologists become more dissimilar during their training.

According to ScanMatch, the least similar scanning patterns were observed on false-negative trials across all within-group comparisons (see Fig. 7). The Global-Focal Search Model postulates that, when viewing a medical image, observers obtain a global impression of the image which constrains subsequent search (Nodine & Kundel, 1987). Similarly, the Two-Stage Detection Model (Swensson, 1980) asserts that experts use visual mechanisms that act as a filter to constrain the features that warrant further examination. Kundel, Nodine, Krupinski, and Mello-Thoms (2008) proposed that the strategy used by radiologists in interpreting medical images consists of a ‘look-detect-scan’ pattern which has a prominent role for a gestalt-like stage of processing. These models fit with the dual-pathway model of visual awareness (Drew, Evans, Võ, Jacobson, & Wolfe, 2013; Wolfe, Vo, Evans, & Greene, 2011) which proposes that search in natural scenes is best explained by two routes: (1) a ‘non-selective’ pathway enables the extraction of global statistical information from within an image (global processing); (2) a ‘selective pathway’ in which individuals select candidate objects for focal processing (Wolfe et al., 2011). It is possible that the lowest scanning similarity was revealed for false negatives because observers did not detect the global irregularities that would drive the subsequent search. Therefore, observers engaged in a less systematic search of the brain and failed to detect the tumour. However, this line of reasoning would also suggest that low scanning similarity would be observed for true negatives because there are also no global irregularities to guide the subsequent search, but this result was not observed. Such findings indicate that models of medical image perception should incorporate the visual search process undertaken when global irregularities are not detected by an observer. Classifying diagnostic accuracy into stimulus-response categories without systematically manipulating the level of conspicuousness of each tumour is a limitation of the present study. It is possible that diagnostic accuracy was affected by the conspicuity of the stimuli and therefore future research should be conducted to address this issue.

For the Expert group, correct diagnosis (i.e. true positives and true negatives) were characterised by higher scanning similarity (see Fig. 7). In line with models of visual search in medical image perception, we postulate that scanning similarity is higher for experts on true-positive trials because experts extract the global properties of an image to constrain the subsequent search in a similar manner to each other. It is possible that high similarity was observed for true-negative trials because experts did not extract global irregularities at the start of their search and instead reverted to a more *default* scanning pattern. More research is required to understand the training processes undertaken by radiologists to explore whether such *default* patterns are taught during training.

Where studies have looked at the similarities or agreement in observers, they have tended to use brain tumour delineation tasks. Low inter- and intra-observer variability is well documented in clinicians delineating brain tumours (Crowe, Alderson, Rossiter, & Kent, 2017; Mazzara, Velthuizen, Pearlman, Greenberg, & Wagner, 2004; Murakami, Hirai, Toya, Nakamura, & Yamashita, 2012; Weltens et al., 2001). This line of research highlights the need to obtain high agreement in tasks relevant to medical image perception to assure consistency both within and between clinicians responsible for making diagnostic decisions. Future research is needed to apply ScanMatch to larger datasets and address questions with clear practical implications. For example, examining intra-observer variability at different timepoints (e.g. first and last case of the day) could provide insight into the effect of internal factors on performance (e.g. fatigue) and comparing scan paths could facilitate monitoring the development of expertise among trainee radiologists. Moreover, identifying scanning patterns that are related to a certain diagnosis could provide insight into why certain diagnostic errors occur and therefore contribute the development of innovative training routines (Krupinski, 2006). A limitation of our study was that we did not obtain any localisation information (i.e. where participants thought the tumour was). These data would have enabled us to use free-responses ROC (FROC) analysis to evaluate observers’ performance which is a statistically more powerful method (Krupinski, 2010). Moreover, examining location information would facilitate differentiating between certain types of error.

This study extends the limited literature examining the effect of expertise and diagnosis on brain tumour detection using MRI images. Both whole-brain and independent-slice brain tumour detection tasks revealed a similar qualitative pattern of results but the effect of viewing modality on gaze behaviour and diagnostic accuracy remains understudied in the literature. We demonstrate the effective application of ScanMatch to the medical interpretation literature and revealed that both expertise level and diagnosis affects the similarity of scanning patterns. Although this is preliminary work, the ScanMatch method is a novel analysis tool that provides insight into both the temporal and spatial components of saccadic eye-movement sequences.

## Conclusion

In summary, this is the first study to apply ScanMatch to the medical image perception literature and suggest that both expertise and diagnosis affect the similarity of scanning patterns in a brain tumour detection task using MRI images. Experts engaged in the most similar scanning patterns for both whole-brain and independent slice viewing modalities and, in the independent-slice viewing task, these patterns were most similar when experts gave a correct diagnosis. We demonstrate the efficacy of using ScanMatch in medical image perception research and so propose that further research is warranted to identify scanning patterns in different presenting medical problems to help increase diagnostic success. Subsequent research adopting this method can address research questions with the potential to inform training practices undertaken by trainee radiologists.

## Abbreviations

CAD: Computer-aided detection
CADe: Computer-assisted detection
CT: Computed tomography
ECG: Electrocardiogram
MRI: Magnetic resonance imaging
NHS: National Health Service
